# Experimental insights into electrocatalytic [Cp*Rh(bpy)Cl]^+^ mediated NADH regeneration

**DOI:** 10.1038/s41598-023-49021-4

**Published:** 2023-12-16

**Authors:** Jonas Meyer, Manuela Romero, Jorg Thöming, Michael Baune, Nicholas Reimer, Ralf Dringen, Ingmar Bösing

**Affiliations:** 1 Chemical Process Engineering Group (CVT), Leobener Strasse 6, 28359 Bremen, Germany; 2 Centre for Biomolecular Interactions Bremen (CBIB), Bremen, Germany; 3 University of Bremen, Bremen, Germany

**Keywords:** Catalysis, Electrochemistry, Environmental chemistry

## Abstract

NADH plays a crucial role in many enzymatically catalysed reactions. Due to the high costs of NADH a regeneration mechanism of this cofactor can enlarge the applications of enzymatic reactions dramatically. This paper gives a thorough system analysis of the mediated electrochemical regeneration of active NADH using cyclic voltammograms and potentiostatic measurements with varying pH, electrode potential, and electrolyte solution, highlighting the system’s limiting conditions, elucidating optimal working parameters for the electrochemical reduction of NAD^+^, and bringing new insight on the oxidation of inactive reduction products. Using [Cp*Rh(bpy)Cl]^+^ as an electron mediator dramatically increases the percentage of enzymatically active electrochemically reduced NADH from 15% (direct) to 99% (mediated) with a faradaic efficiency of up to 86%. Furthermore, investigations of the catalytic mechanisms of [Cp*Rh(bpy)Cl]^+^ clarifies the necessary conditions for its functioning and questions the proposed reaction mechanism by two-step reduction where first the mediator is reduced and then brought in contact with NAD^+^.

## Introduction

Unconventional production pathways must be established to maintain the chemical supply in the future and achieve net zero CO_2_ emissions by 2050 as planned by the European Green Deal^1^. Enzymes are likely to play an important role in alternative production processes since they catalyse complex reactions under ambient conditions and exhibit high activity and selectivity^2^. About one-fourth of all enzymatic reactions are oxidoreductases which depend on coenzymes such as NADH or NADPH^3^. NAD^+^ and its reduced counterpart NADH enable several enzymatic reactions which can, among others, fixate CO_2_ into valuable chemical products. The electrochemical regeneration of NADH for such reactions would offer an alternative storage for surplus renewable energy which could be exploited for the production of valuable chemicals whenever the conventional storage capacities are full. Despite the great potential, a major hurdle remains since the high cost of NADH makes it more expensive than the chemicals it produces^3^. This makes its use in wide-scale manufacture commercially unprofitable without a proper regeneration cycle of the consumed NADH.

The quest for an in situ NADH regeneration method to circumvent this bottleneck in enzyme-based synthesis has caused a wide range of NADH regeneration strategies (chemical, enzymatic, photocatalytic, electrochemical (mediated and direct)) which are a prominent topic of studies over the last four decades^4^. Among the investigated methods, mediated electrochemical reduction of NAD^+^ with [Cp*Rh(bpy)Cl]^+^ (2,2ʹ-bipyridyl)(pentamethyl-cyclopentadienyl)-rhodium has proven to deliver the highest selective conversion towards the enzymatically active 1,4-NADH^5^. In contrast to direct regeneration, mediated NAD^+^ reduction requires lower electric potentials which in turn results in higher faradaic efficiencies since one of the major contributors to loss of faradaic efficiency, the concurring hydrogen evolution reaction (HER), is slower at less cathodic potentials. The high selectivity towards active NADH from this organometallic mediator additionally prevents the loss of active 1,4-NADH into the enzymatically inactive NAD^+^ reduction products: NAD_2_, 1,2-NADH and 1,6-NADH.

The electrochemical NAD^+^ reduction (direct and mediated) is intensively studied and the effect of pH^6–9^, electrode potential^6,10–15^, temperature^7,10,11,14,16,17^, electrode material^10,13,15,18,19^, mediator/cofactor concentrations^6,8,9,14^, and mediator types^5,9,20,21^, have been reviewed in the literature.

Furthermore, there are numerous proofs of concept for mediated and direct bio-electrochemical NAD^+^/NADH-based synthesis that yield a variety of chemical compounds, including: l-glutamate^22^, methanol^20,23,24^, formaldehyde^24^, lactate^6,11,25^, formate^24,26,27^, d-sorbitol^28^, triose phosphate and 3-phosphoglycerate (3GPA)^29^, Formic Acid^21,24^. These studies have demonstrated the feasibility of joining electrochemical regeneration with enzymatic production, a logical next step would be the systematic optimization of the regeneration to be able to consider the design of larger scale production.

In a previous study^6^ it was observed how decreasing pH until a value of 7 led to an increase in the percentage of active species in direct NADH regeneration using a copper electrode. Further decrease of pH values to 6.5 resulted in a decreased percentage of active NADH due to hydrogen evolution reaction occurring at less cathodic potentials. This was also the case NADH regeneration mediate by the [Cp*Rh(bpy)Cl]^+^ complex where the electrocatalytic reduction of NAD^+^ was more effective at lower pH values^9^. Hollmann et al.^7^ and Walcarius et al.^9^ investigated the effect of pH on the electrocatalytic behaviour of the Rh-mediator and NAD^+^ cofactor couple. Walcarius et al. used cyclic voltammetry to determine the best pH range for the mediated regeneration. They found, based on the peak current of CVs, the pH range between 6.5 and 7.5 to be most effective for the NADH regeneration. The pH dependency is explained by the proton availability for the protonation Rh-complex. Hollman et al.^7^ also evaluated the activity of this rhodium complex for a wide pH range (3.8–8.5) and found out it displayed maximum activity at a pH of 7. Contrary to the other studies Azem et al.^8^ reported that on a Ruthenium functionalized glassy carbon electrode the reduction of NAD^+^ is pH independent for a pH range between 5.5 and 10.2 and justified this with the myriad of protons absorbed in Ru sites at the electrode surface which support the reduction of NAD^+^ into NADH. Based on these works we studied the actual 1,4-NADH yield, faradaic efficiency and production rate of the mediated NADH regeneration on different pH values to shed light on these differences and to investigate if yield and efficiency follow the same trends.

As pointed out by Saba et al., many publications report NADH generation without discerning between inactive and enzymatically active species which imposes “an extreme challenge in understanding the reaction mechanism for further optimization”^30^. Furthermore, important quantitative data such as faradaic efficiency, selectivity, turn-over frequency, end concentrations or specific production rates of both active and inactive species required for the materials balance equations have not always been presented, making a comparison among different studies and parameters extremely difficult.

This paper presents a comprehensive study of the [Cp*Rh(bpy)Cl]^+^ mediated electrochemical NADH regeneration. The papers objective is to highlight which experimental conditions result in the highest selectivity, yield, and faradaic efficiency of 1,4-NADH after a systematic optimization of pH, electrical potential, mediator state and buffer system. Additionally, insight about the oxidation of inactive NADH species is presented. Many studies have set focus on the reduction of NADH, but since no method with a 100% selectivity towards active NADH has been reported, the recycling of inactive species through their oxidation into NAD^+^ remains relevant, particularly when considering a continuous production scenario since otherwise inactive species accumulate.

## Methods

### Materials

Trishydroxymethyl aminomethan (TRIS), pyruvate, HCl and NaOH were purchased from Sigma-Aldrich (Taufkirchen, Germany) and 4-(2-hydroxyethyl)-1-piperazineethanesulfonic acid (HEPES), NAD, NADH, and lactate dehydrogenase (LDH) from ITW reagents (Darmstadt, Germany). Chemicals used for the research were used without further purification. The [Cp*Rh(bpy)Cl]^+^ rhodium complex was synthesized by Hansa Fine Chemicals (Bremen, Germany) using 2,2ʹ-bipyridyl and the pentamethylcyclopentadienylrhodium(III)-chloride dimer that had been purchased from Sigma Aldrich following methods described in literature^5^.

As working electrodes glassy carbon electrodes, a rod (2 mm diameter), and a plate (4 cm^2^), were used. The measurements were conducted using a Ag/AgCl reference electrode and a platinum counter electrode (2 cm diameter, 0.1 mm thick). The potentiostat used was a Metrohm Autolab PGSTAT204 with the Nova software package. All experiments were carried out at room temperature. A three-electrode cell was used in all experiments. If not stated otherwise 0.1 M Tris/HCl buffer pH (TRIS) and HEPES/NaOH buffer pH 7.0 (HEPES buffer) were used for the measurements.

During the regeneration measurements, the counter electrode was separated from working and reference electrode by a PEM membrane (Nafion-117). The Nafion membrane absorbs larger amounts of [Cp*Rh(bpy)Cl]^+^. As a result, the [Cp*Rh(bpy)Cl]^+^ concentration would decrease during the experiments. To prevent this, the membrane was saturated in advance in a high molar [Cp*Rh(bpy)Cl]^+^ solution.

### Cyclic voltammetry and potentiostatic measurements

Prior to each measurement a glassy carbon rod was wet polished to mirror finish using 1 µm diamond paste and thereafter sonicated in ethanol. The glassy carbon electrode was pre-treated using a 0.5 M H_2_SO_4_ solution for 10 cycles at a scan rate of 0.1 V/s in a potential window between −1.5 and 1 V. All measurements were carried out in an oxygen-free solution, which was purged for at least 10 min with N_2_ before each measurement. A N_2_ atmosphere above the solution’s surface was ensured during all the measurements. Each measurement was repeated at least three times.

### Electrochemical regeneration of NADH

The glassy carbon plate electrode was prepared exactly like the rod electrode for the cyclic voltammetry measurements. The electrolyte was vigorously purged with N_2_ and a constant N_2_ atmospheric flow was ensured during the regeneration. Before the reduction potential was set, a cyclic voltammogram was taken, thereafter the potentiostatic measurement was carried out for 1800 s at room temperature. After regeneration a cyclic voltammogram was recorded using the glassy carbon plate electrode and thereafter the solutions for the enzyme assay were prepared.

### Calculation of active NADH

To calculate the active NADH concentrations an enzyme assay with pyruvate as educt and lactate as product was used.

1 $${\text{pyruvate}} + 1,4{\text{-NADH}}\mathop \to \limits^{{{\text{LDH}}}} {\text{lactate}} + {\text{NAD}}^{ + }$$

The concentration of 1,4-NADH (in a 0.8 diluted solution) is calculated by UV–Vis extinction at 340 nm (${\text{Ext}}_{{1,4{\text{-NADH}},340\;{\text{nm}}}}$) using the following equation.

2 $$c_{{1,4{\text{-NADH}}}} = \frac{{\frac{{{\text{Ext}}_{{1,4{\text{-NADH}},340{\text{nm}}}} }}{{0.8}}-b_{{{\text{NADH}},340{\text{nm}}}} }}{{m_{{{\text{NADH}},340{\text{nm}}}} }}$$

${m}_{\text{NADH},340\text{ nm}}$ and ${b}_{\text{NADH},340\text{ nm}}$ are the slope and the offset of the UV–Vis calibration curve of 1,4-NADH prepared at different concentrations. The 340 nm wavelength is used due to the extinction peak of 1,4-NADH (Supplementary Material Fig. 1). Due to the dilution of the solutions in Table 1, the dilution factor 0.8 must be considered.

**Table 1:** Solutions measured for calculation of active enzyme substrate 1,4-NADH. “buffer” refers to the same buffer solution used in the experiment (0.1 M TRIS or HEPES) at the corresponding pH value. “pyruvate” refers to a 50 mM pyruvate solution in the corresponding buffer. “LDH” refers to a 1 U (enzyme's catalytic activity) LDH solution in the corresponding buffer.

	Solution
A	80% electrolyte + 20% buffer
B	80% regenerated solution + 20% buffer
C	80% regenerated solution + 10% pyruvate + 10% buffer
D	80% regenerated solution + 10% pyruvate + 10% buffer; after measurement of E
E	80% regenerated solution + 10% pyruvate + 10% LDH

The extinction of 1,4-NADH at a wavelength of 340 nm is calculated by measuring the extinction of the solutions containing 1,4-NADH (D) minus the extinction of the solution without 1,4-NADH (E), minus the extinction of the Rh-complex itself (compare Table 1):

3 $${\text{Ext}}_{{1,4{\text{-NADH}},340{\text{ nm}}}} = {\text{D}}_{{340{\text{ nm}}}} - {\text{E}}_{{340{\text{ nm}}}} - \Delta {\text{Ext}}_{{{\text{Rh-complex}},340{\text{ nm}}}}$$

The extinction of the Rh-complex can change in between solutions D and E and is compensated by $\Delta {\text{Ext}}_{{{\text{Rh-complex}},340{\text{ nm}}}}$.

4 $$\Delta {\text{Ext}}_{{{\text{Rh-complex}},340{\text{ nm}}}} = \left( {\Delta c_{{{\text{Rh-complex}}}} \cdot m_{{{\text{Rh-complex}},340{\text{ nm}}}} + b_{{{\text{Rh-complex}},340{\text{ nm}}}} } \right) \cdot 0.8$$

The slope $m$ and the offset $b$ are calculated from the UV–Vis calibration curve at 340 nm wavelength of the electrolyte solution at different concentrations. Spectroscopic examination of the electrolyte to establish a calibration curve is performed for each experiment. These measurements are made as close in time as possible to the measurements in Table 1.

The extinction changes of the Rh-complex in the solutions D (solution before enzymatic NADH reduction, containing active 1,4-NADH) and E (solution after enzymatic reduction, containing no 1,4-NADH) is determined from the extinction at 430 nm using the 430 nm calibration curve of the electrolyte solution. At 430 nm wavelength only the Rh-complex shows extinction (Supplementary Material Fig. 1).

5 $$\Delta {c}_{\text{Rh-complex}}=\frac{\frac{{\text{D}}_{430\text{ nm}}-{\text{E}}_{430\text{ nm}}}{0.8}-{b}_{\text{Rh-complex},430\text{ nm}}}{{m}_{\text{Rh-complex},430\text{ nm}}}$$

The selectivity was calculated by the fraction of absorbance at 340 nm given by active 1,4-NADH divided by the absorbance at 340 nm given by NAD_2_ and all NADH species. The Faraday efficiency ${\eta }_{\text{F}}$ was calculated by the theoretically charge necessary for the 1,4-NADH regeneration ${Q}_{\text{theo}}$ divided by the total charge during the regeneration ${Q}_{\text{total}}$:

6 $${\eta }_{\text{F}}=\frac{{Q}_{\text{theo}}}{{Q}_{\text{total}}}$$

Thus, the selectivity is affected only by inactive NAD-species, whereas the Faraday efficiency can also be affected by the HER (and other side reactions).

## Results and discussion

### Cyclic voltammograms

**Figure 1:**
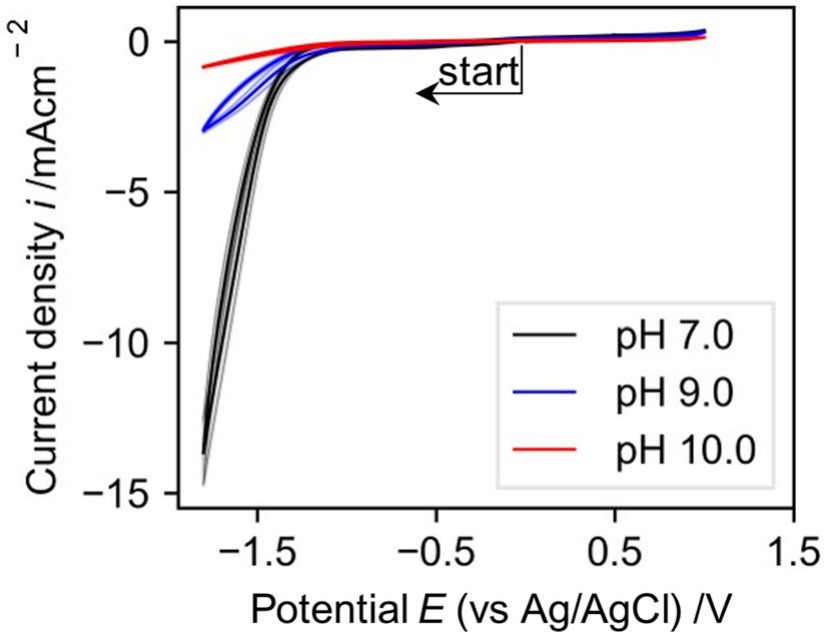
Cyclic voltammograms of GC electrodes in Tris buffer to establish the electrochemical window of the TRIS buffer solution. CVs recorded at a scan rate of 0.1 V/s in 0.1 M TRIS at different pH values (0 V → −1.8 V → 1.0 V → 0 V). Shaded area indicates standard deviation of 3 different CVs.

The selected buffer plays an important role in both, the electrochemical characterization of the system and the reduction of NAD^+^. For electrochemical characterization by potential sweeps buffer without reduction peaks in the range where the investigated substances are reduced/oxidized is desired. TRIS, particularly at higher pH values, has a large potential range without reduction or oxidation peaks (Fig. 1) making it a good candidate for electrochemical characterizations of the substances used.

Additionally, for the reduction of NAD^+^, a buffer in which both, products and educts, are stable for a long time is also desirable. Although phosphate buffer has been a popular choice for the regeneration of NADH across many studies^7–9,12,13,15–17,19,21,28,31–47^ TRIS buffer was preferred because NADH has been demonstrated to be unstable in phosphate buffer^6,30,48^. This instability could lead to an overestimation of active 1,4-NADH, since the measuring method employed assumes that the enzymatic oxidation of 1,4-NADH is the only cause for the decreasing absorption at 340 nm. A simultaneous decay of inactive species (1,6- or 1,2-NADH and NAD_2_) due to their instability in phosphate buffer, would lead to a larger decrease in the absorption compared to that caused by the enzyme-catalysed reaction alone and therefore the amount of active NADH could be overestimated^6^. It has been reported that the selectivity of the rhodium complex as mediator towards the reduction of active NADH in TRIS is lower than that observed in diammonium hydrogen phosphate buffer^7^. However, in our experiments the regeneration in TRIS has yielded rhodium complex-mediated 1,4-NADH regenerations with a selectivity up to 99%.

#### Cyclic voltammetry of NAD^+^

**Figure 2:**
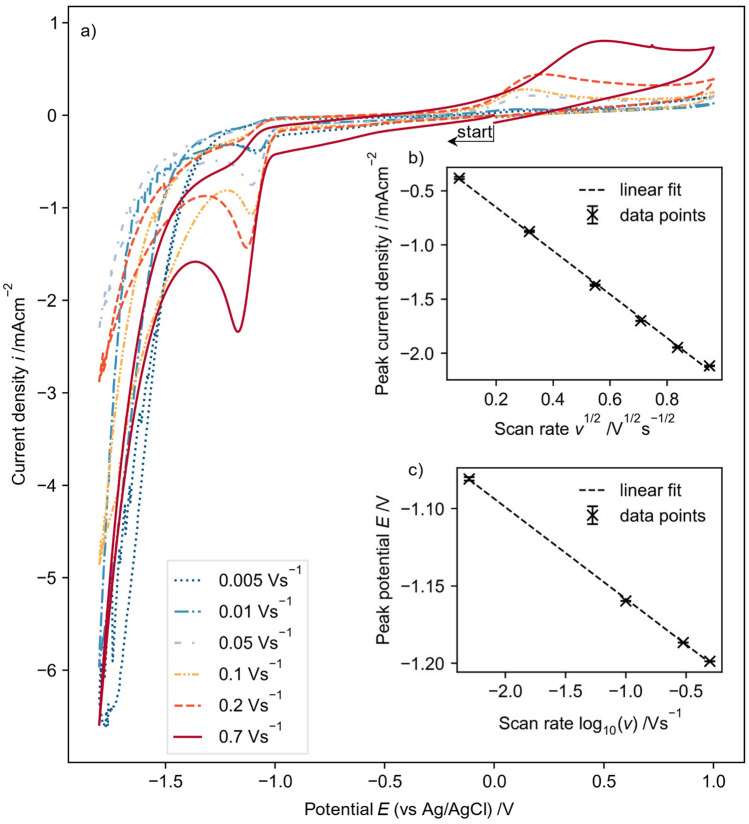
Electrochemical characterization of NAD^+^ performed by (**a**) recording cyclic voltammograms using a GC rod electrode with 5 mM NAD^+^ in 0.1 M TRIS at pH7.0 across various scan rates with a potential curve of (0 V → −1.8 V → 1.0 V → 0 V). Analyzing the first reduction peak reveals (**b**) the dependence of the peak current to the scan rate and (**c**) the dependence of the peak potential on the scan rate.

Cyclic voltammograms were carried out at different scan rates starting in cathodic direction to establish the kinetics of NAD^+^ reduction (Fig. 2). With increasing scan rate, the reductive peak potentials become more negative, whereas a dependency of the peak potential on the scan rate indicates irreversible behaviour for the given scan rates^49^. This suggests that NAD^+^ reduction is an electrochemically irreversible reaction, thus, the reaction kinetic is rather slow compared to the mass transport. However, the clearly pronounced reduction peaks show a diffusion control for sufficient high overpotentials.

**Figure 3:**
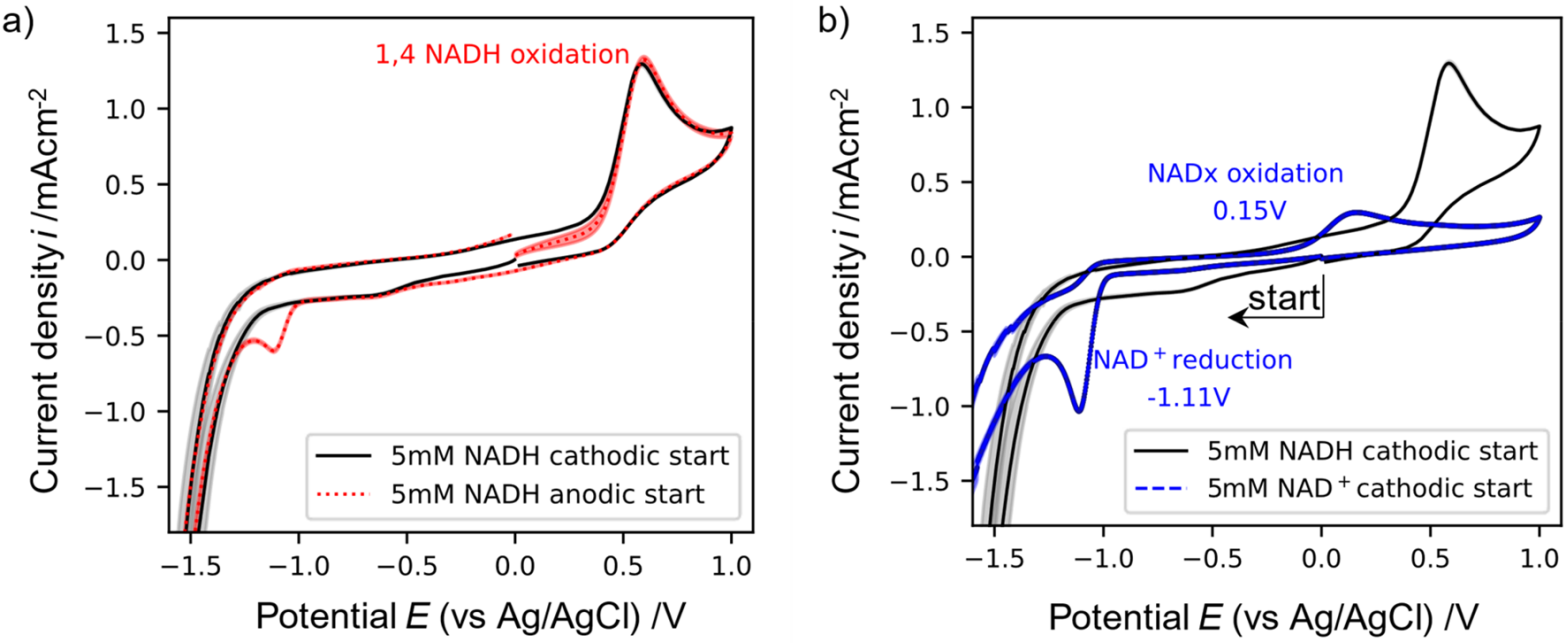
Cyclic voltammograms recorded with a GC rod electrode at a scan rate of 0.1 V/s, all solutions contain 0.1 M TRIS buffer at pH 7.0. (**a**) 5 mM 1,4-NADH with cathodic and anodic start direction. (**b**) 5 mM NAD^+^ and 5 mM 1,4-NADH starting in cathodic direction. Shaded area indicates standard deviation of 3 different CVs.

The cathodic scan starts at 0 V with a low capacitive current. At around −1.0 V a reduction reaction is visible, which shows a reductive peak between −1.08 and −1.18 V, depending on the scan rate. The reductive peak can be associated to the reduction of NAD^+^. The reduction peak is followed by an increasing cathodic current from −1.4 V due to hydrogen formation. At −1.8 V the scan direction is reversed and the cathodic current decreases. At ca. −1.0 V the current is slightly anodic and shows a constant capacitive current up to 0.0 V. At 0.1–0.5 V (depending on the scan rate) an anodic peak due to the oxidation of the NADH/NAD_2_ species is visible. Compared to the oxidation of pure 1,4-NADH the peak occurs at lower anodic potential (see Fig. 3b), which indicates that the inactive species (probably mostly NAD_2_) shows a lower oxidation potential. That phenomenon can be used to oxidize inactive NADH/NAD_2_ species to NAD^+^ without oxidizing 1,4-NADH.

A dependency of peak potential ${E}_{\text{p}}$ and scan rate of an irreversible reaction is given by Nicholson and Shain^49^:

7 $${E}_{\text{p},2}-{E}_{\text{p},1}=-\frac{RT}{\alpha nF}(\text{ln}\left(\sqrt{{\nu }_{2}}\right)-\text{ln}\left(\sqrt{{\nu }_{1}}\right))$$

with the universal gas constant $R$, the absolute temperature $T=293$ K, the scan rate $\nu$ and charge transfer coefficient $\alpha$ and number of transferred electrons $n$. Rearranging leads to:

8 $$\frac{\text{d}E}{\text{dln}(\nu )}=-\frac{1}{2}\frac{RT}{\alpha nF}$$

And for the decade logarithm we found:

9 $$\frac{\text{d}E}{\text{dlog}(\nu )}=-2.303\times \frac{1}{2}\frac{RT}{\alpha nF}=-\frac{0.029}{\alpha n}$$

With the given dependency (Fig. 2 inlet c) this leads to $\alpha n$ = 0.49.

#### Cyclic voltammetry of NADH

Electrochemical characterization of pure 1,4-NADH and its comparison to NAD^+^ and electrochemically generated reduction products (NADH and NAD_2_) can uncover differences in their electrochemical behaviour. These variations can be valuable for re-oxidizing inactive NADH species and NAD_2_ back to NAD^+^ or in differentiating active NADH from inactive NADH. To investigate these differences in the electrochemical behaviour CVs of pure 1,4-NADH and NAD^+^ are performed in TRIS buffer. Figure 3a shows a CV of pure enzymatically active 1,4-NADH examined first in cathodic direction and then a new sample examined starting in anodic direction. In both cases the oxidative peak of NADH has its maximum at 0.6 V. It can also be observed that for the scan which started in anodic direction, a small amount of NADH which was oxidized is later reduced since a reductive peak is visible at −1.1 V. This peak coincides with the reduction peak usually observed for NAD^+^ and suggests that at potentials higher than 0.6 V enzymatically active NADH is oxidized into NAD^+^.

Figure 3b shows CVs of pure 1,4-NADH and NAD^+^ started in cathodic scan direction. After the reduction of NAD^+^ observed at −1.1 V, a slight oxidative peak can be observed in that same solution around 0.15–0.25 V. This suggests that the produced species at −1.1 V (mixture of NAD_2_, 1,2-NADH, and 1,6-NADH) can be oxidized at lower potentials than the authentic 1,4-NADH which is oxidized at 0.6 V (visible from the CV of pure 1,4-NADH). It should be highlighted that direct reduction of NAD^+^ with glassy carbon did not deliver high percentages of active NADH as will be discussed later (see Fig. 11 and Supplementary Fig. 2). Considering the low regeneration rates of active 1,4-NADH at glassy carbon (compare “Direct NADH regeneration”) and the different oxidation peak potential compared to pure 1,4-NADH it seems plausible that the species oxidized between 0.15 and 0.25 V in these CVs are mainly the inactive NAD_2_ dimer and 1.2 and 1.6 isomers.

The separation of both oxidation peaks, inactive NAD species around 0.15 V and active 1,4-NADH at 0.6 V, enables an electrochemical way to distinguish both species. Furthermore, it enables NAD^+^/NADH reduction–oxidation cycles in which NAD^+^ will be reduced to different NAD species (inactive NAD_2_ and NADH and active 1,4-NADH). Afterwards the inactive species will be oxidized back to NAD^+^ at low anodic potentials at which the active 1,4-NADH does not react.

#### Cyclic voltammetry of [Cp*Rh(bpy)Cl]^+^

**Figure 4:**
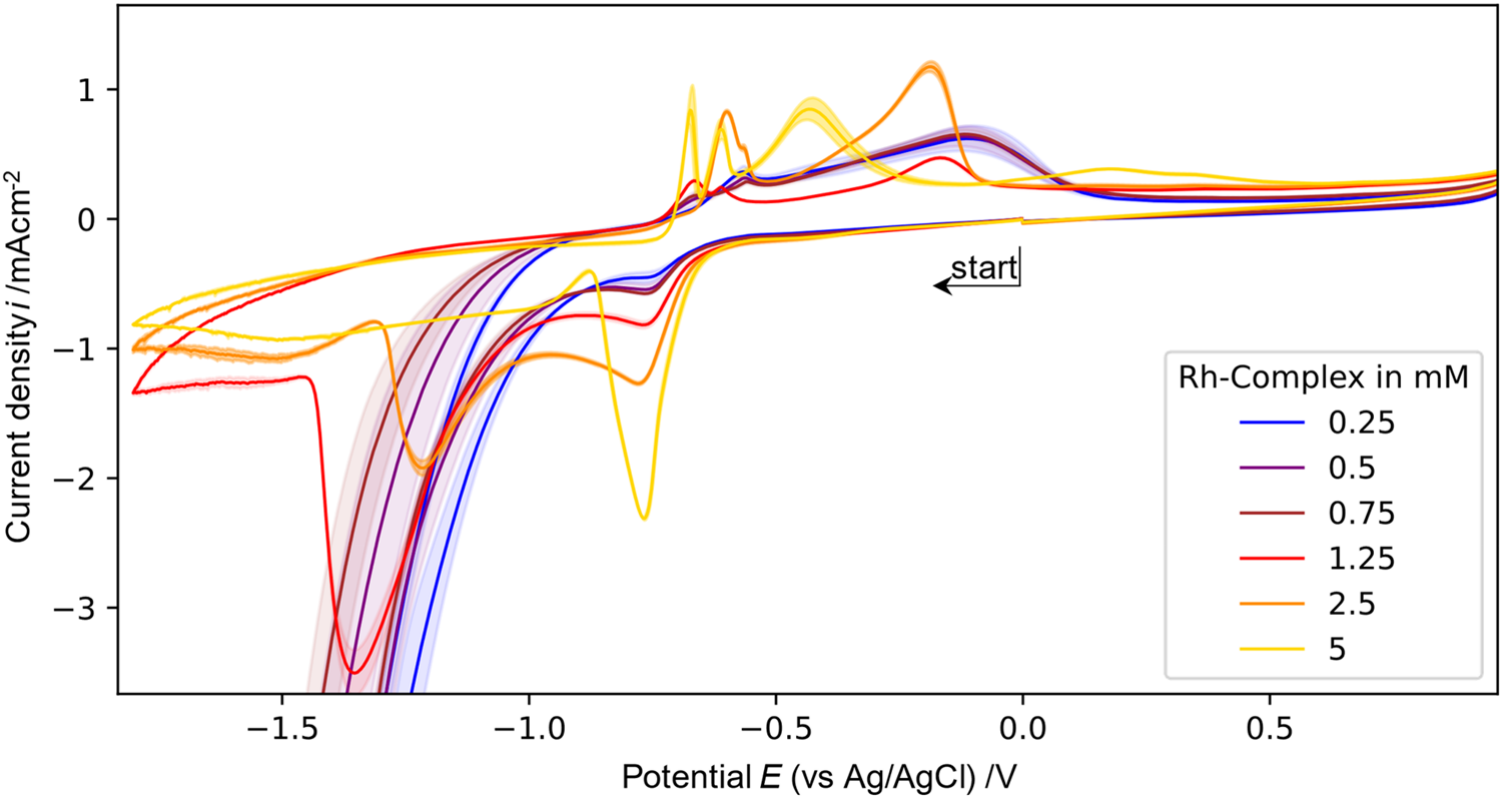
Cyclic voltammograms recorded with a GC rod electrode with different concentrations of [Cp*Rh(bpy)Cl]^+^ in 0.1 M TRIS at pH 7.0 at a scan rate of 0.1 V/s (recorded from 0 V → −1.8 V → 1.0 V → 0 V). Shaded area indicates standard deviation of 3 different CVs.

Cyclic voltammetry of the [Cp*Rh(bpy)Cl]^+^ rhodium complex reveals the electrochemical behavior of this electron mediator. At −0.6 V the reduction current increases and at −0.75 V a first reduction peak can be observed, followed by a second broad peak between −1.0 and −1.5 V, depending on the concentration (Fig. 4). A scan in anodic direction after both reduction peaks are completed leads to a sharp oxidation peak at −0.6 V followed by a broader peak at −0.4 V. The reduction peaks are associated to the reduction of Rh(III) to Rh(II) and Rh(I) and possibly to Rh(0). As can be seen from the regeneration measurements (below) and the evaluation of the kinetic parameters there is probably already Rh(I) present during the first reduction peak and the peaks result from the overlay of several reduction reactions. Thus, both peaks cannot separately attributed to the reduction of Rh(III) and Rh(II). Furthermore, the near absence of the second reduction peak for high concentrations but the presence of several oxidation peaks indicates that several reductions contribute to the first peak. The anodic peaks are accordingly attributed to the oxidation of the rhodium ions.

A strong dependency of the CV shape on the concentration of the Rh-complex was observed. While the first reduction peak is more pronounced at higher concentrations, the second reduction peak decreases with increasing [Cp*Rh(bpy)Cl]^+^ concentration. At very low rhodium complex concentrations the CV at cathodic potentials is dominated by the hydrogen formation. The absence of hydrogen formation for high concentrations indicates a strong adsorbance of the rhodium complex on the electrode surface. A properly pronounced second reduction peak is only visible at 2.5 mM and 1.25 mM of the complex. This indicates that the surface is not fully blocked by the rhodium complex at lower concentrations, thus, hydrogen evolution reaction can take place. Nevertheless, the current decreases after further cathodic scan (for concentrations above 0.75 mM) which indicates a later adsorbance at the electrode surface and blocking of the surface. This electrode blocking takes place at less cathodic potentials (earlier) the higher the concentration (Fig. 4). Furthermore, with increasing concentration of rhodium complex the hydrogen evolution reaction (HER) is hindered and the high currents observed at −1.5 V in pure TRIS buffer at pH 7.0 are only visible when the complex concentration is lower than 1.25 mM. This information is crucial since HER is a concurring reaction that decreases the Faradaic efficiency of the process. Knowing the minimum rhodium complex concentration to hinder this process is valuable since the electric current can be redirected through the correct concentration of mediator to the reduction of NAD^+^ into active 1,4-NADH.

**Figure 5:**
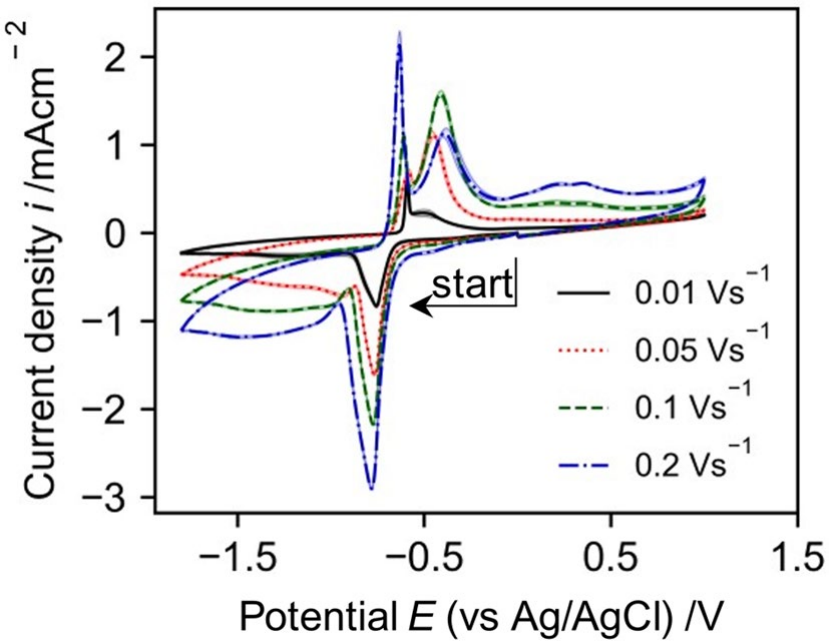
Cyclic voltammograms recorded with a GC rod electrode with 5 mM [Cp*Rh(bpy)Cl]^+^ in 0.1 M TRIS at pH 7.0 at different scan rates (recorded from 0 V → −1.8 V → 1.0 V → 0 V). Shaded area indicates standard deviation of 3 different CVs.

**Figure 6:**
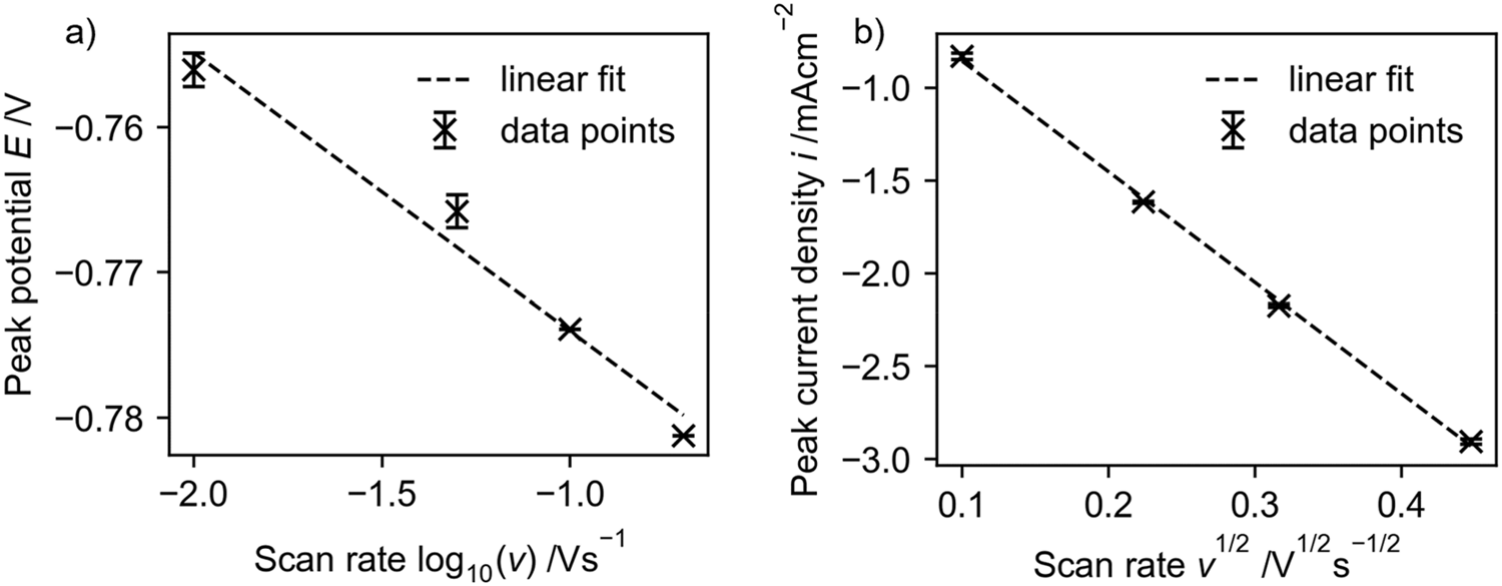
Characterization of the reduction peaks observed in the CVs of [Cp*Rh(bpy)Cl]^+^ in Fig. 5. (**a**) The dependence of peak potential on scan rate and (**b**) the dependence of peak current on scan rate.

The effect of scan rate on the reduction and oxidation peaks is as expected. With increasing scan rate the peak currents increase and the peak potentials of the reduction reaction shift to more cathodic potentials (Fig. 5). This indicates irreversible behavior of the [Cp*Rh(bpy)Cl]^+^ reduction reaction. Using the presented equations for the dependency of peak potential (a) and peak current (b) on the scan rate (above) leads to an $\alpha n$-product of $\alpha n=1.51$. This indicates that more than 1 electron is transferred during this reduction step (since $\alpha <1$) (Fig. 6).

#### Cyclic voltammetry of [Cp*Rh(bpy)Cl]^+^ in combination with NAD^+^

**Figure 7:**
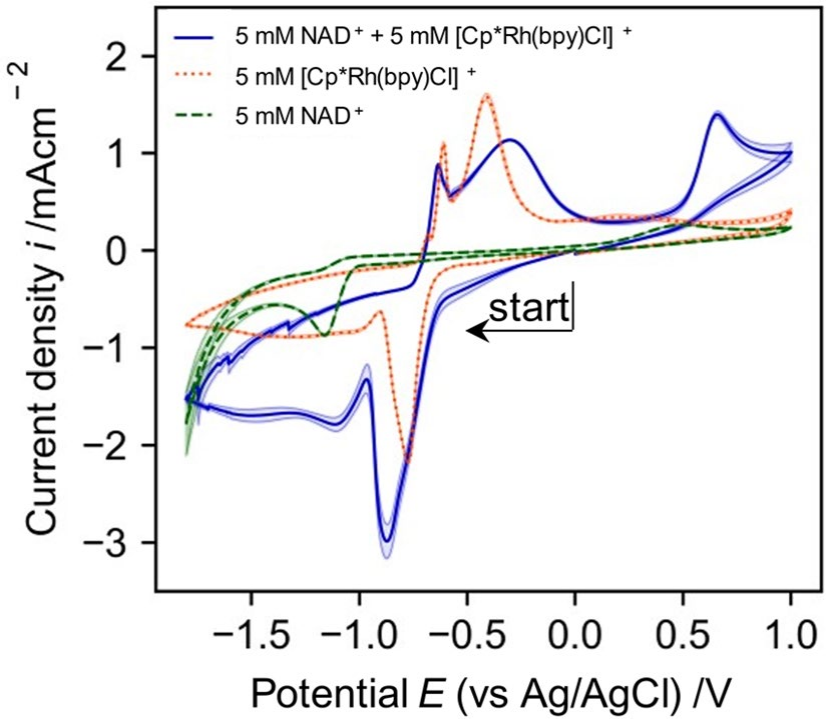
Cyclic voltammograms of glassy carbon electrode in 0.1 M TRIS buffer at pH 7.0 with a scan rate of 0.1 Vs^−1^ with NAD^+^ and rhodium complex separated and together (recorded from 0 V → −1.8 V → 1.0 V → 0 V). Shaded area indicates standard deviation of 3 different CVs.

A CV of the [Cp*Rh(bpy)Cl]^+^ Rhodium complex in presence with NAD^+^ clarifies the interplay between both species (Fig. 7). During a cathodic scan the reduction peak attributed to the Rhodium complex is more pronounced in presence of NAD^+^. This is because the electrochemically reduced Rhodium complex is directly oxidized by the NAD^+^ and can be reduced additional times.

At the end of the anodic scan a clearly pronounced oxidation peak at around 0.6 V is visible. This oxidation peak corresponds to the oxidation of 1,4-NADH and clarifies that the oxidation potential of the active 1,4-NADH species is more anodic compared to that of the inactive NAD_2_ species (oxidation peak around 0.2 V, Fig. 3b).

Cyclic Voltammetry of the different electrochemically active species investigated, NAD^+^, NADH and [Cp*Rh(bpy)Cl]^+^ delivers valuable insight into their electrochemical behavior and interplay. The reduction of NAD^+^ starts at around −1.0 V, which defines the electrochemical window for the mediated NADH regeneration. The regeneration potential should be more anodic than −1.0 V to avoid the direct reduction of NAD^+^ which leads to mostly inactive NAD-species, as visible from the CVs. However, the inactive reduction product of NAD^+^ reduction at GC electrodes can be oxidized at lower anodic potentials compared to the oxidation potential of 1,4-NADH, as visible from the CVs. The reduction of the [Cp*Rh(bpy)Cl]^+^ mediator in presence of NAD^+^ is followed by fast formation of active NADH as visible from the more pronounced reduction peak of [Cp*Rh(bpy)Cl]^+^ and the additional oxidation peak at 0.6 V compared to CVs of pure [Cp*Rh(bpy)Cl]^+^. To gain additional insights into the electrochemical [Cp*Rh(bpy)Cl]^+^ reduction at GC electrodes, that go beyond the findings from the CVs, impedance measurements are performed.

### Impedance measurements

Electrochemical impedance (EIS) measurements can be used to distinguish faradaic from capacitive currents, and thus to learn more about the electrochemical system. A comparison of EIS measurements of GC electrodes in absence and presence of [Cp*Rh(bpy)Cl]^+^ can deliver additional insights into the reduction mechanism of the mediator.

**Figure 8:**
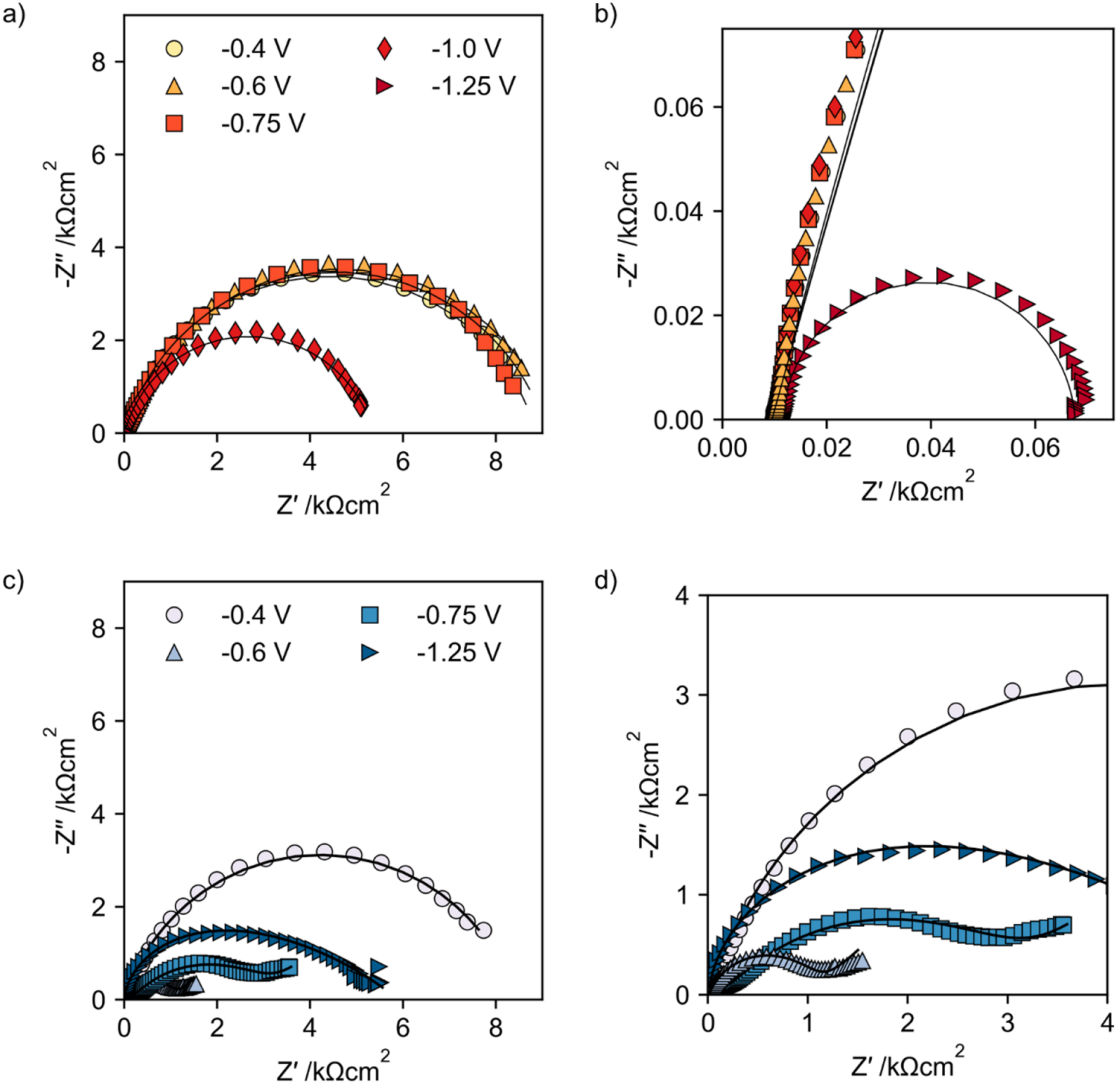
Nyquist plots of glassy carbon electrodes after reduction at different cathodic potentials (**a**) & (**b**) in TRIS buffer and (**c**) & (**d**) in TRIS buffer and 5 mM [Cp*Rh(bpy)Cl]^+^ complex. Marker represent data and lines the equivalent electrical circuit fits given in Fig. 9.

The impedance measurements of glassy carbon electrodes emerged in TRIS buffer without and with addition of 5 mM [Cp*Rh(bpy)Cl]^+^ show a strong dependency of the electrochemical behavior on the electrode potential (Fig. 8). At low cathodic potential (−0.4 V vs Ag/AgCl) the impedance spectra in TRIS buffer with and without [Cp*Rh(bpy)Cl]^+^ complex are similar and dominated by the capacitive behavior of the double layer and a weak cathodic reaction.

**Figure 9:**
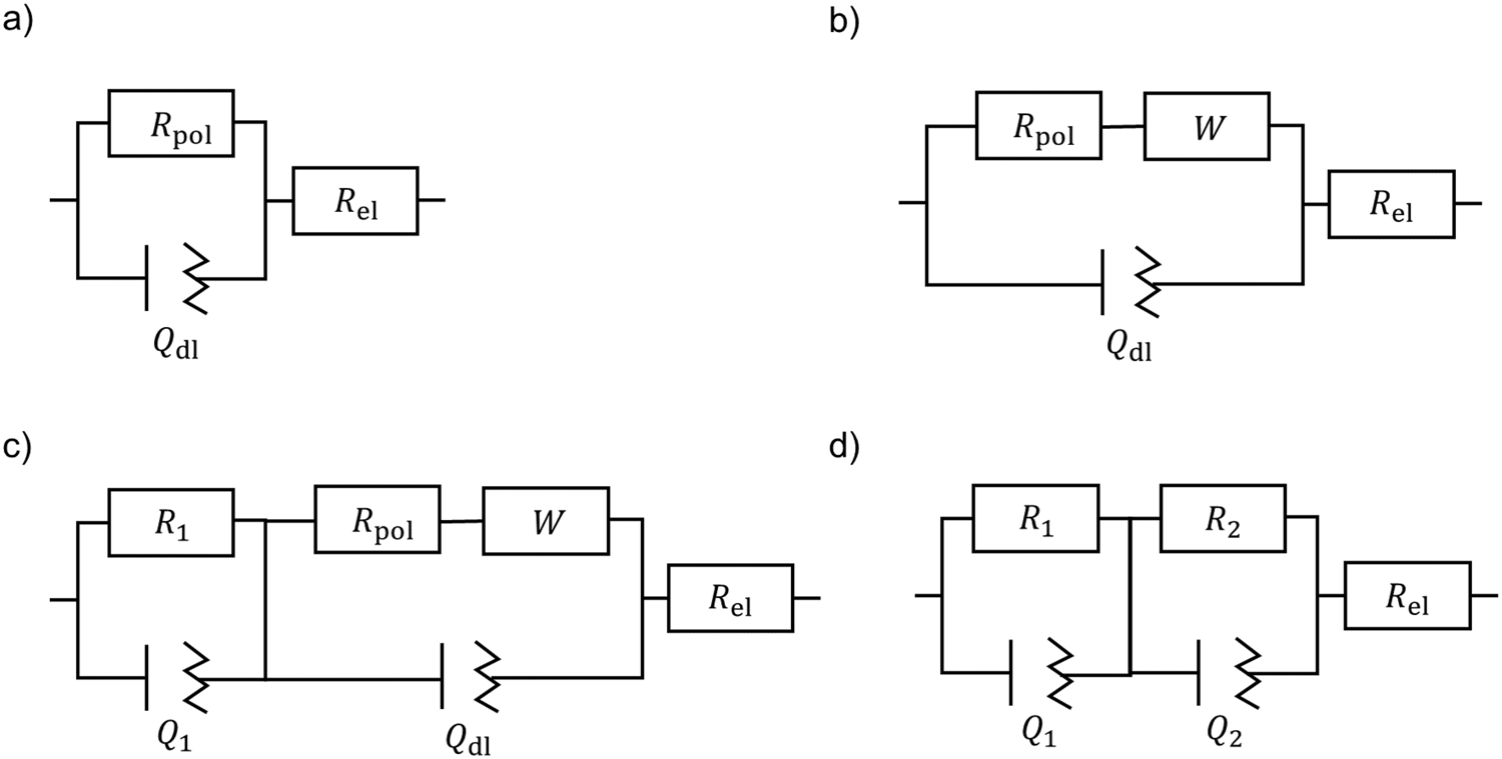
Equivalent electrical circuits (EECs) to fit the impedance measurements for glassy carbon electrodes in TRIS buffer with and without addition of [Cp*Rh(bpy)Cl]^+^ complex. (**a**) Pure TRIS buffer all potentials and TRIS + 5 mM [Cp*Rh(bpy)Cl]^+^ −0.4 V, (**b**) TRIS + 5 mM [Cp*Rh(bpy)Cl]^+^ −0.6 V (**c**) TRIS buffer + 5 mM [Cp*Rh(bpy)Cl]^+^ −0.75 V (**d**) TRIS buffer + 5 mM [Cp*Rh(bpy)Cl]^+^ − 1.0 V and﻿ −1.25 V.

With increasing cathodic potential (−0.6 V, −0.75 V, −1.0 V) the impedance behavior of the glassy carbon electrode in TRIS buffer without addition of the rhodium complex shows no significant differences (Fig. 8a). In contrast the impedance of the glassy carbon electrode in TRIS buffer with addition of the [Cp*Rh(bpy)Cl]^+^ complex significantly changes due to the accelerated cathodic reaction, the adsorption of the rhodium complex on the electrodes surface and the formation of an adsorbed rhodium complex film at the electrodes surface (Fig. 8c,d). At −0.6 V the impedance behavior is characterized by diffusion of [Cp*Rh(bpy)Cl]^+^ towards the electrode, which can be seen by a linear branch of the impedance at low frequencies and the necessity of a Warburg element in the EEC to fit the impedance data (Fig. 9b). Further increasing the cathodic potentials leads to formation of a rhodium complex film at the electrode surface (second time constant necessary in EEC), whereas this formation seems to be under diffusion control, as can be seen by the necessity of a Warburg element to represent the impedance behavior (Fig. 9c). At −1.25 V the film is completely formed at the electrodes surface and the impedance can be represented by a dual layer, given by the capacitive behavior of the double layer and the adsorbed rhodium complex (Fig. 9d).

While the total impedance without addition of the rhodium complex decreases with increasing cathodic potential, due to the accelerated hydrogen formation reaction, the impedance in the solution with addition of [Cp*Rh(bpy)Cl]^+^ decrease by changing the potential from −0.4 to −0.6 V but increases with more cathodic potentials due to the formation of the adsorbed rhodium complex layer.

From the EECs which are fitted to the impedance data (Fig. 9) it is possible to derive parameter values for the polarization resistance and to calculate the capacity by^50^:

$${C}_{\text{eff}}=\frac{{\left({Y}_{0}{R}_{\text{p}}\right)}^\frac{1}{n}}{{R}_{\text{p}}}\text{sin}\left(\frac{n\pi }{2}\right).$$

**Figure 10:**
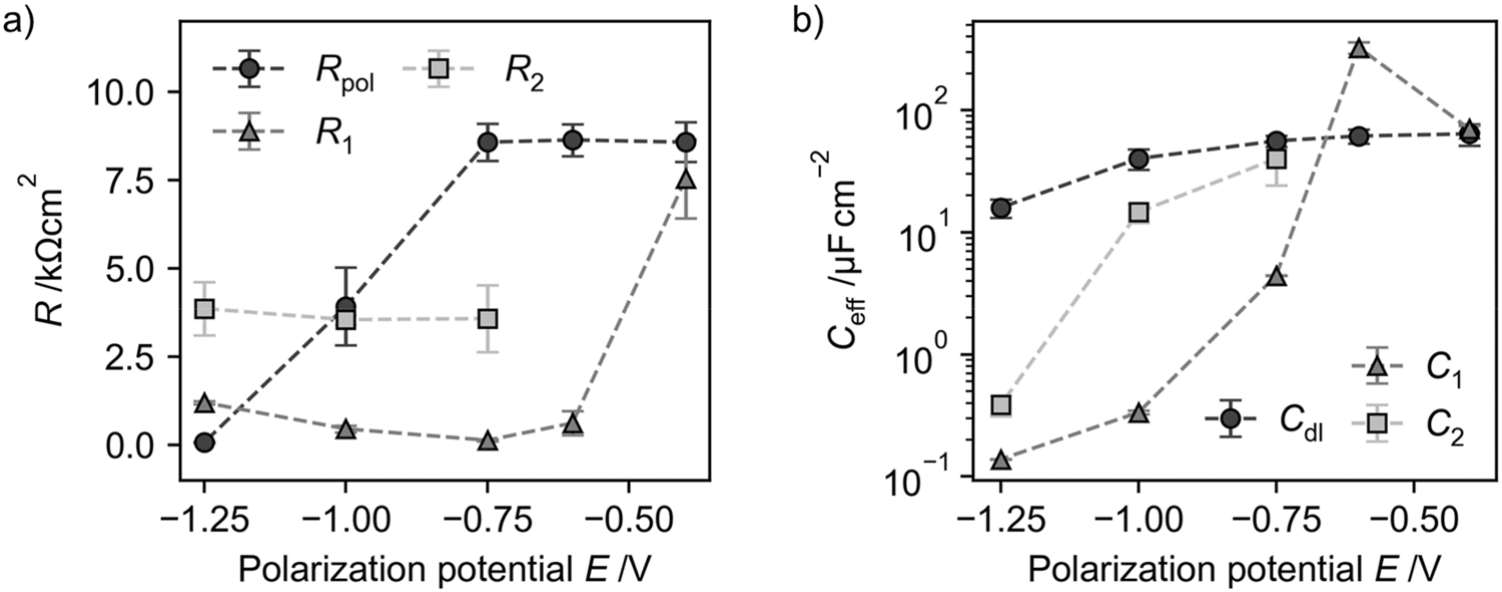
Dependencies of the (**a**) resistances and (**b**) capacities on the electrode potentials, calculated from the EECs given in Fig. 9.

The polarization resistance ${R}_{\text{pol}}$ in pure TRIS buffer is nearly constant at low cathodic potentials (−0.4 V, −0.6 V and −0.75 V) (Fig. 10a), due to the absence of an increasing cathodic reactions. After onset of the hydrogen evolution reaction (−1.0 V) the polarization resistance decreases as expected with increasing cathodic potential.

The EEC resistances in the presence of [Cp*Rh(bpy)Cl]^+^ first decrease with more cathodic potentials, due to the onset of Rh-complex reduction. With further increasing of the cathodic potential the resistances remain nearly constant, supporting the hypothesis that no additional reduction of the [Cp*Rh(bpy)Cl]^+^ complex takes place at more cathodic potentials but the reduction from Rh(III) to Rh(II) and to Rh(I) take place at nearly the same potentials.

The double layer capacity in pure TRIS buffer is in the expected range and lies between 10 and 50 µF/cm^2^ (Fig. 10b). The capacities in presence of the Rh-complex decrease with increasing cathodic potentials, indicating a more pronounced, thicker adsorbed layer at more cathodic potentials and less electrode area at which the double layer can form.

### NADH regeneration

Chronoamperometric experiments are used to study NADH regeneration on a glassy carbon electrode with and without electron mediator. The catalytic abilities of the [Cp*Rh(bpy)Cl]^+^ as well as the direct NADH regeneration are investigated under variation of pH values and electrode potential.

#### Direct NADH regeneration

**Figure 11:**
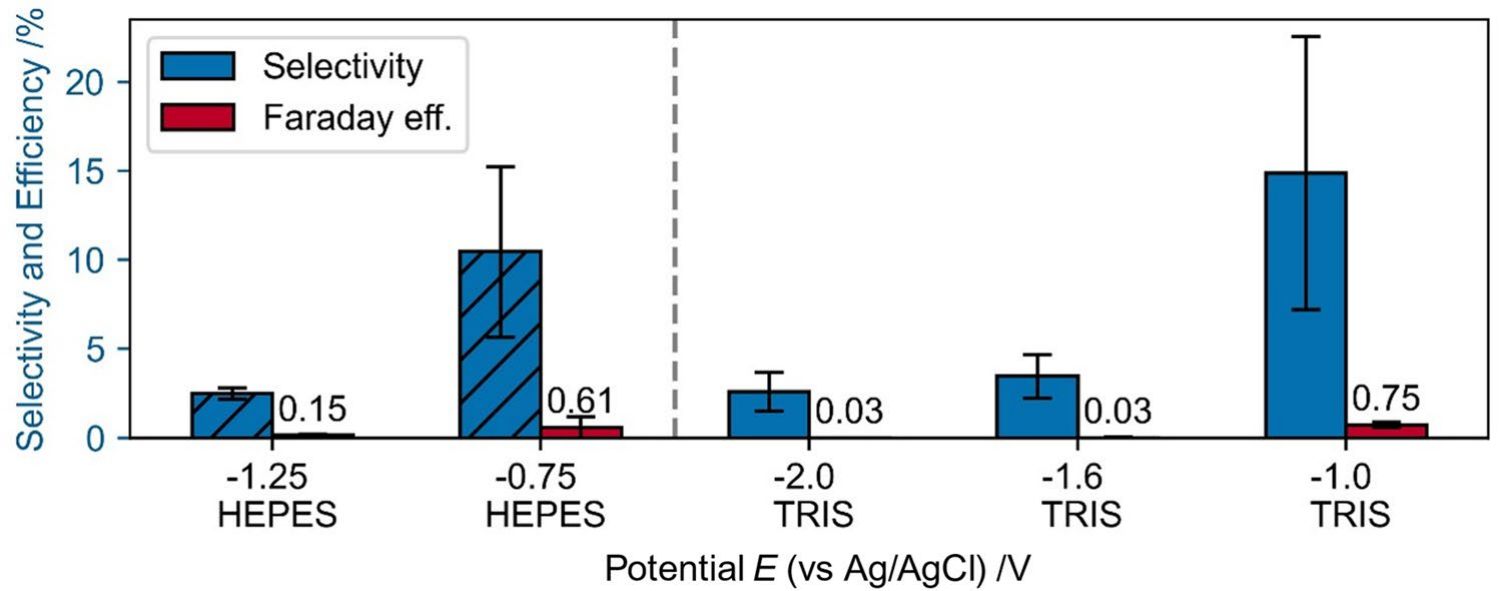
Direct NADH regeneration for 1800 s using a glassy carbon electrode in absence of rhodium based mediator. Regeneration solutions with 1 mM NAD^+ ^in 0.1 M HEPES and in 0.1 M TRIS buffer.

The direct regeneration of NADH using a glassy carbon electrode at pH 8 in HEPES and TRIS buffer leads to a comparable low selectivity towards 1,4-NADH (Fig. 11). The selectivity and faradaic efficiency decrease as more cathodic potentials are applied in both buffer systems, possibly due to the accelerated hydrogen formation. In general, higher selectivities are achieved using TRIS as a buffer. The selectivity is highest at −1 V vs Ag/AgCl in TRIS buffer. The high selectivities towards 1,4-NADH at glassy carbon electrodes at high cathodic potentials reported elsewhere^36^ could not be confirmed by us. Due to the very low extinctions after direct NADH regeneration at glassy carbon (compare Supplementary Fig. 2) it is possible that even these low selectivities are only artefacts from the measurement technique and we recommend to handle these values with care.

#### Mediated NADH regeneration

**Figure 12:**
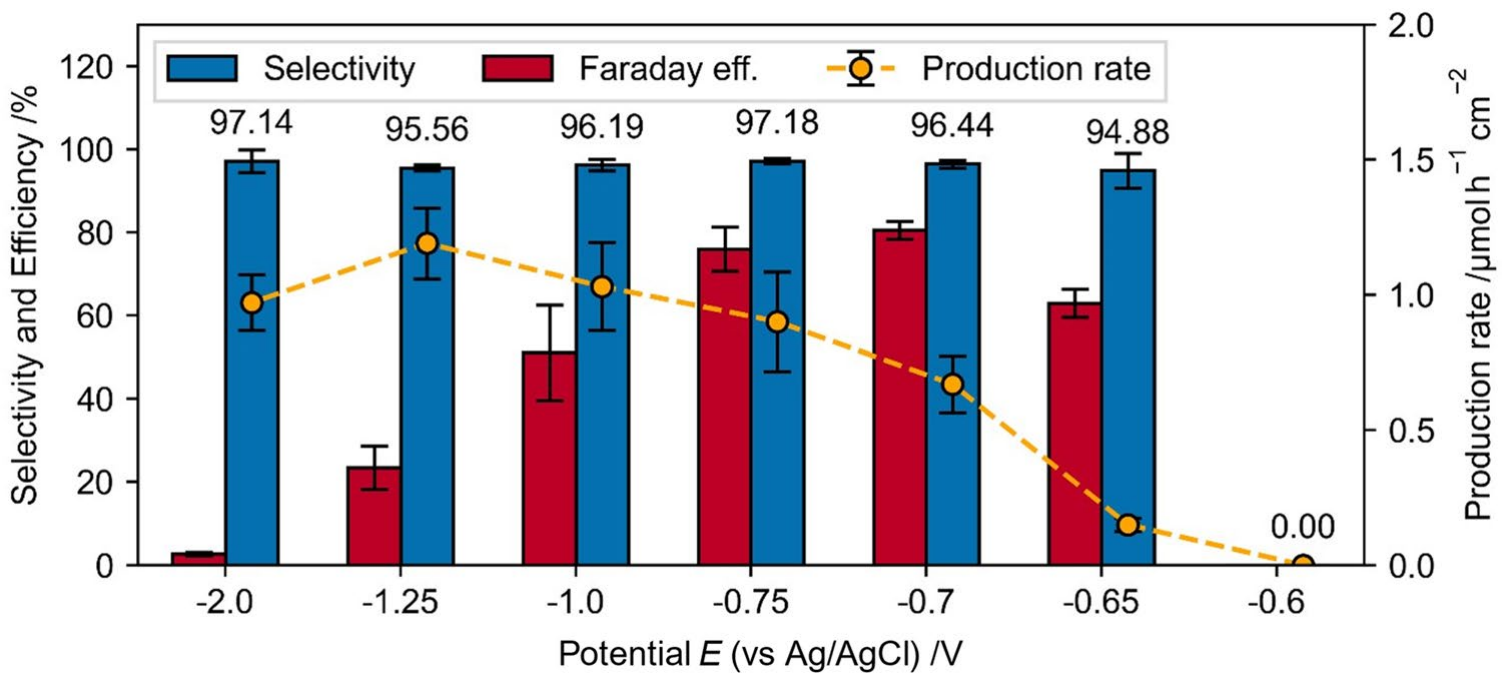
Potential dependence of Faradaic efficiency, selectivity and production rate of the mediated NADH regeneration with 0.25 mM [Cp*Rh(bpy)Cl]^+^ + 1 mM NAD^+^ + 0.1 M TRIS at pH 8.0, for 1800 s.

Compared to the direct NADH regeneration, the production rate, selectivity and Faraday efficiency of the mediated regeneration are significantly higher (Fig. 12). Whereas the selectivity shows very high values for a broad range of experimental parameters, faradaic efficiency and specific production rate show a strong dependency on the experimental conditions.

The potential dependent NADH regeneration in TRIS buffer containing [Cp*Rh(bpy)Cl]^+^ reveals partly opposing dependencies of the selectivity, faradaic efficiency and production rate on the electrode potential, which indicates that compromises between these variables must be made in order to apply and optimize the process (Fig. 12).

With onset of the rhodium complex reduction (−0.65 V) 1,4-NADH recovery is possible with very high selectivity, which underlines that at this potential the reduction to Rh(I) takes place (not only Rh(II)) and the reduced complex [Cp*Rh(bpy)Cl]^+^ is present in the solution. With increasing reduction potential, the selectivity increases slightly, shows a maximum at −0.75 V and decreases afterwards. The Faraday efficiency has a maximum at −0.7 V (80 %) and decreases with increasing reduction potential, whereas the production rate increases up to a cathodic potential of −1.25 V and decreases with further increasing of the reduction potential.

**Figure 13:**
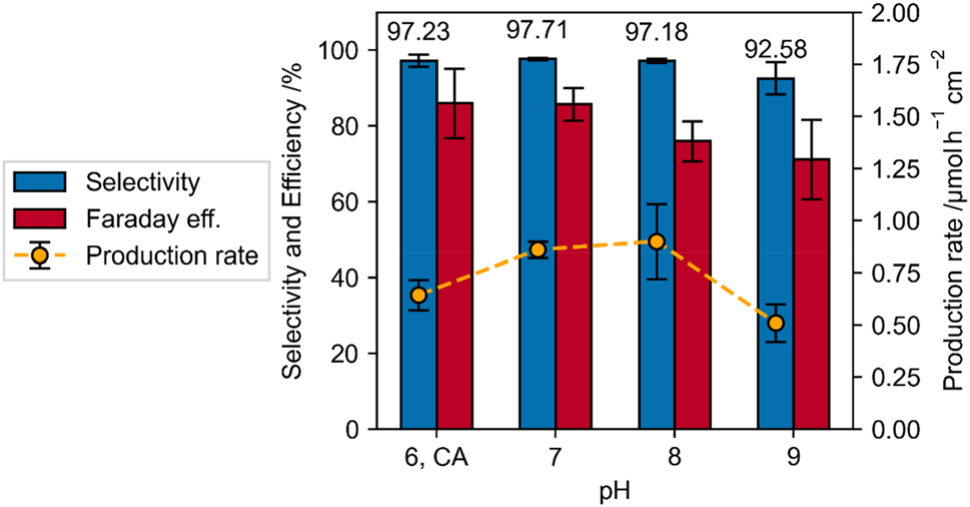
pH dependence of Faradaic efficiency, selectivity and production rate of the mediated NADH regeneration with 0.25 mM [Cp*Rh(bpy)Cl]^+^  + 1 mM NAD^+^ for 1800 s at −0.75 V vs Ag/AgCl in 0.1 M TRIS buffer at different pH values, pH = 6 is performed in citric acid (CA).

The pH value is expected to have a direct impact on the NADH regeneration, due to the protonation of the [Cp*Rh(bpy)Cl]^+^ complex^9^. Figure 13 shows the selectivity, Faraday efficiency and specific production rate during the NADH regeneration using the rhodium complex in TRIS buffer as electron mediator at different pH values. It can be observed that regenerations carried out at pH 7 possess the highest selectivity compared to measurements carried out at different pH values. This finding agrees with^9^, however the differences between pH 6, 7 and 8 regarding the 1,4-NADH selectivity are neglectable small and all pH values seem to work equally fine for the NADH regeneration with respect to the selectivity. Further increasing the pH value to pH 9 leads to a significant loss in 1,4-NADH selectivity. This can partially be explained because the concentration of hydrogen ions at lower pHs is considerably higher and these are essential for the rhodium complex reduction and consequent production of NADH. The specific production rate at pH 7 and 8 is significantly higher compared to the rate at pH 6 and 9. This agrees with the results of Hollman et al. who found out maximum activity of the rhodium complex for the NAD^+^ reduction at pH 7^6,7^. It can be noted, that the Faraday efficiency is increasing with decreasing pH, showing its maximum at pH 6, even though it is expected that the hydrogen evolution reaction (HER) would decrease with increasing pH. Thus, the HER is not the factor for decreasing Faraday efficiencies, which also fits with the observation of the blocked electrode surface by the [Cp*Rh(bpy)Cl]^+^ complex.

**Figure 14:**
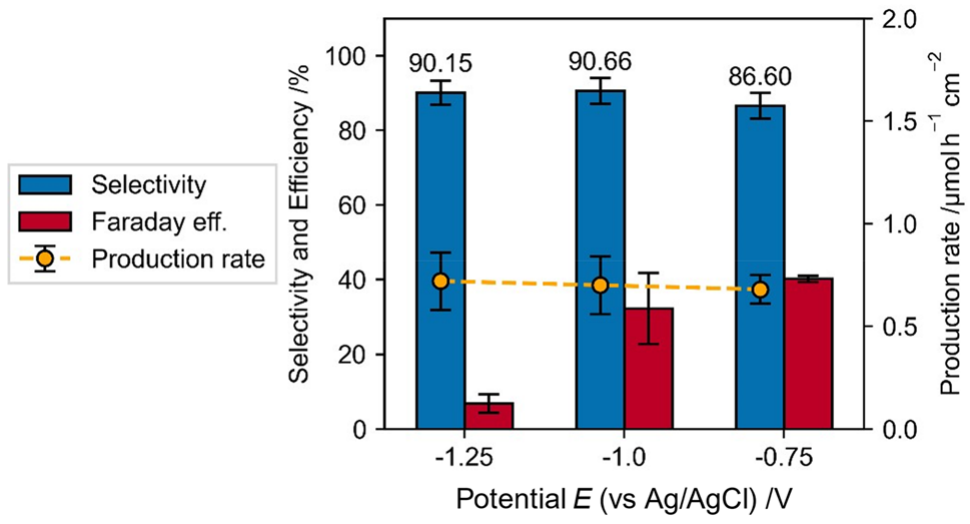
Mediated NADH regeneration in 0.1 M HEPES buffer at pH 8.0 with 0.25 mM [Cp*Rh(bpy)Cl]^+^ + 1 mM NAD^+^, for 1800 s.

The mediated 1,4-NADH regeneration is also possible in HEPES buffer, even though selectivity, efficiency and production rate are significantly lower compared to the regeneration in TRIS buffer (Fig. 14). In contrast to the NAD^+^ reduction in TRIS buffer, the production rate is nearly potential independent for the tested potentials. The Faraday efficiency depends on the potential as demonstrated by increasing efficiency with more anodic electrode potentials. Due to the decreasing HER with more anodic potential this behavior is expected. In an ongoing study we detected that Cl^-^ ions can prevent the oxidation of NADH to NAD^+^ which might explain the lower selectivities, because the pH value of TRIS is adjusted with HCl, while the pH of chloride-free HEPES had been adjusted with NaOH. A publication including this and further observation regarding the NADH oxidation is currently in preparation.

#### Two step reduction of NAD^+^ using [Cp*Rh(bpy)Cl]^+^ and time dependency

**Scheme 1.**
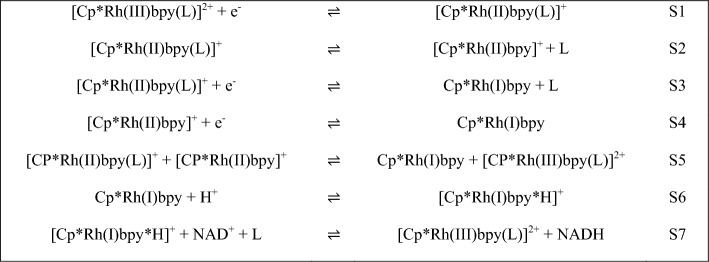
Reaction mechanism of [Cp*Rh(bpy)Cl]^+^ reduction and NADH regeneration proposed by Hildebrand et al.

Gaining insight into the reaction mechanism that governs the interaction between the Rhodium-complex and NAD^+^ holds the potential to inspire novel experimental configurations and enhance our comprehension of the inherent constraints of the system. Hildebrand et al. proposed a reaction mechanism for the reduction of the [Cp*Rh(bpy)Cl]^+^ complex followed by the reduction of NAD^+^ and re-oxidizing of the [Cp*Rh(bpy)Cl]^+^ complex^5^ (Scheme 1).

As can be seen, reaction steps S1, S3 and S4 are electrochemical reductions, which can occur at the electrode surface, whereas reaction step S2, S5, S6 and S7 are no electrochemical reactions, which can happen anywhere inside the solution.

To test if this allows a two-step reaction procedure, first the reduction of the [Cp*Rh(III)(bpy)] complex by an external potential, followed by the chemical reaction between the reduced [Cp*Rh(I)(bpy)] complex and NAD^+^ without external potential to form [Cp*Rh(III)(bpy)] and NADH, two different experimental procedures are applied and compared to the batch rhodium complex reduction and NADH regeneration.

In the first case, the Rh-complex was reduced 10 min in the absence of NAD^+^ and after 10 min NAD^+^ was added to the solution, while the potential was still hold at a reduction potential. In the second case, the Rh-complex was reduced for 30 min. Afterwards the reduction was stopped and NAD^+^ was added to the solution.

**Figure 15:**
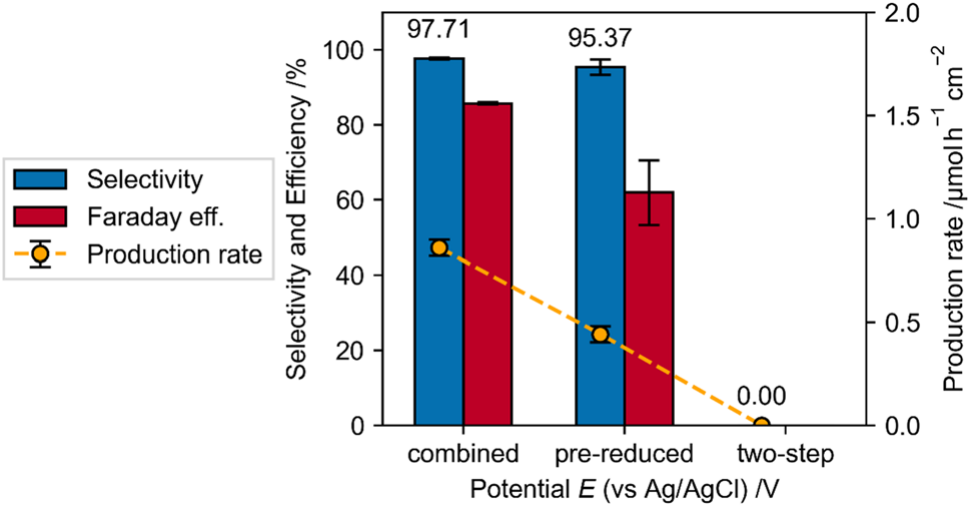
Comparison between dissolved and immobilized rhodium complex (pH 7, −0.75 V). Combined: batch reduction of rhodium complex and NAD^+^ in solution, pre-reduced: rhodium complex is reduced for 10 min and then NAD^+^ is added to the solution, while the potential is still applied, two-step: rhodium complex is reduced for 30 min, reduction is stopped and NAD^+^ is added to the solution.

In the first case (pre-reduced), the selectivity was comparable to the previous experiments with combined complex reduction and NADH regeneration (Fig. 15), while the specific production rate and Faraday efficiency was significantly lower (which can be explained by the 10 min reduction in absence of NAD^+^). In the second case (two-step), prior [Cp*Rh(III)(bpy)] reduction followed by NAD^+^ insertion without external potential, no active NADH was found. This indicates that the proposed reaction mechanism is either not correct or the reduced [Cp*Rh(I)(bpy)] oxidizes back to [Cp*Rh(III)(bpy)] and is not available for the NADH regeneration. The latter seems unlikely since this would also happen during the combined batch reduction and regeneration in the bulk phase of the electrolyte. Thus, we conclude that the NADH regeneration only happens at the electrode surface adsorbed [Cp*Rh(I)(bpy)] species.

**Figure 16:**
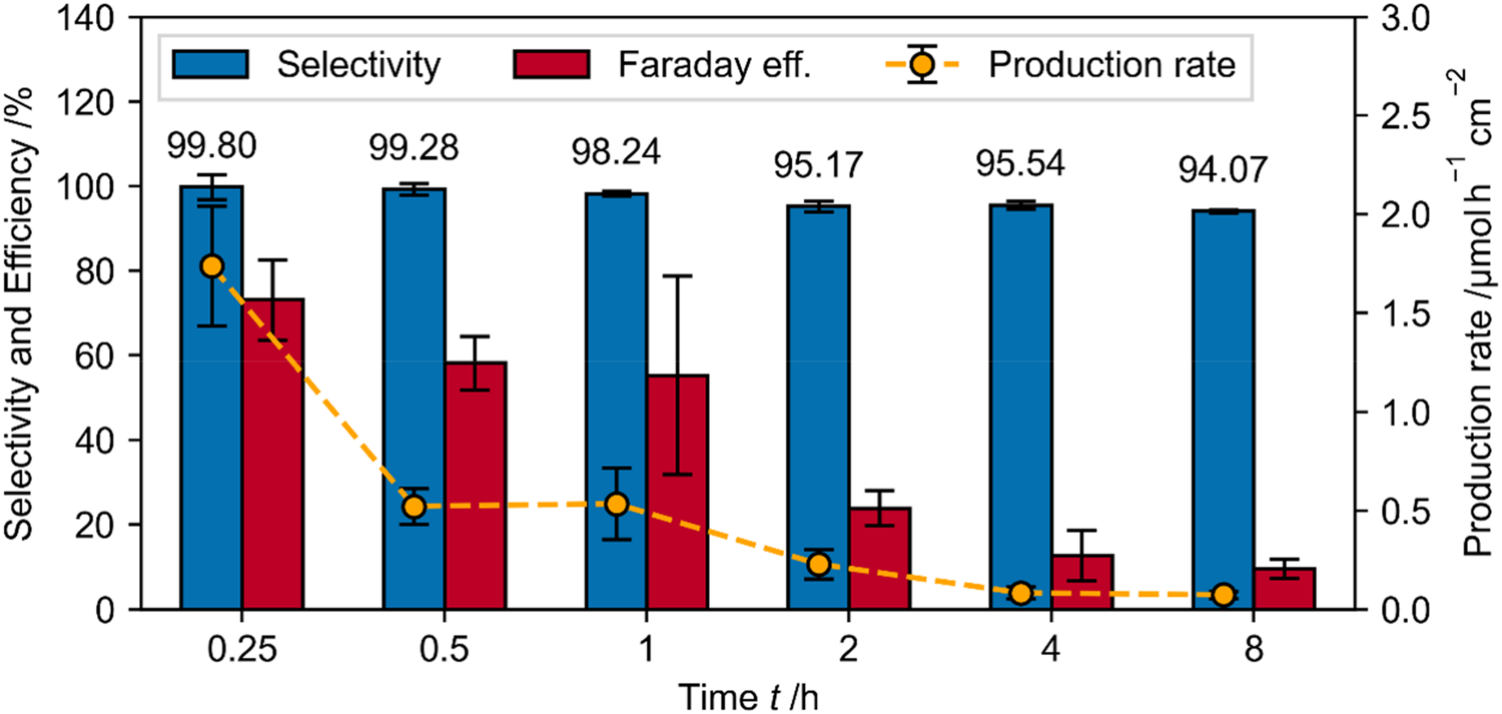
Selectivity, Faraday efficiency and production rate regarding 1,4-NADH during mediated electrochemical NADH recovery over time. Regeneration of 0.25 mM [Cp*Rh(bpy)Cl]^+^ + 1 mM NAD^+^ + 0.1 M TRIS at pH 7, at − $0.75 \text{ V}$ vs Ag/AgCl. The values are described during the specific interval, thus production rate at 4 h is the production rate between 2 and 4 h (not 0–4 h).

To regenerate NADH in large quantities or in a closed loop, the long-term behavior of the experimental setup is crucial. The selectivity, Faraday efficiency and production rate decrease with increasing regeneration time (Fig. 16) indicating that a higher NAD^+^ concentration is beneficial for 1,4-NADH recovery. Furthermore, with ongoing reduction time the regenerated 1,4-NADH can be oxidized by [Cp*Rh(bpy)Cl]^+^ back to NAD^+^, which decreases selectivity, Faraday efficiency and production rate. Furthermore, the GC electrode can change its surface properties and thus its electrochemical activity. Further experiments are planned in a flow cell with the aim of immobilizing the Rh-complex onto the electrode surface. This could enhance the NADH regeneration's long-term efficacy by maintaining high NAD^+^ levels while limiting the Rh complex to the electrode surface only.

## Conclusion

A comprehensive study of experimental parameters on the electrochemical NADH regeneration at glassy carbon is given. The main findings are.

- The direct electrochemical regeneration of NADH at glassy carbon leads to very poor selectivities (< 15 %) and faradaic efficiencies (< 1 %) at all cathodic potentials (considering the very low extinction values the found NADH could also be only measurement artefacts). In comparison shows the rhodium complex mediated regeneration high selectivities (95–99 %) and Faraday efficiencies (up to 86 %).
- With onset of the [Cp*Rh(bpy)Cl]^+^ reduction, 1,4-NADH regeneration is possible with very high selectivity. This indicates that either [Cp*Rh(III)(bpy)] is reduced directly to [Cp*Rh(I)(bpy)] even at low cathodic overpotentials or that the disproportionation of 2 [Cp*Rh(II)(bpy)] leads to a [Cp*Rh(III)(bpy)] and a [Cp*Rh(I)(bpy)] species of which the latter can reduce the NAD^+^.
- Although the selectivity of the rhodium complex-mediated 1,4-NADH regeneration is nearly potential independent, Faraday efficiency and production rate show a high dependency on the reduction potential. This is especially important for possible applications of this procedure and the optimization of the regeneration process regarding energy consumption and NADH yield.
- The highest 1,4-NADH selectivity during mediated regeneration could be found for pH 7 (as already discussed by^7,9^. However, the highest faradaic efficiency was found at pH 6, which also shows a comparable high selectivity.
- The reduction of [Cp*Rh(bpy)Cl]^+^ happens after adsorption of the complex on the electrode surface. Some findings indicate that the subsequent reduction of NAD^+^ also takes place at the adsorbed species.
- The adsorption of [Cp*Rh(bpy)Cl]^+^ at the electrode surface suppresses the hydrogen formation reaction, which increase the Faraday efficiency. Higher [Cp*Rh(bpy)Cl]^+^ concentrations increase this effect.
- The buffer system can play an important role for the electrochemical NADH regeneration. Our findings demonstrated that the selectivity towards 1,4-NADH is significantly higher in TRIS buffer compared to HEPES buffer.

### Supplementary Information

Supplementary Figures.

## Data Availability

The datasets generated and/or analysed during the current study are available in the “Experimental insights into electrocatalytic [Cp*Rh(bpy)Cl]^+^ mediated NADH regeneration DATA” repository, https://doi.org/10.5281/zenodo.8296821.
