# Supplementary material for: Experimental insights into electrocatalytic [Cp*Rh(bpy)Cl]+ mediated NADH regeneration
Source: Sci Rep. 2023 Dec 16;13:22394. doi: 10.1038/s41598-023-49021-4 (PMC10725497; doi:10.1038/s41598-023-49021-4)
Supplement: Supplementary file 1 — Supplementary Figures. [file 41598_2023_49021_MOESM1_ESM.docx]

Supplementary Material


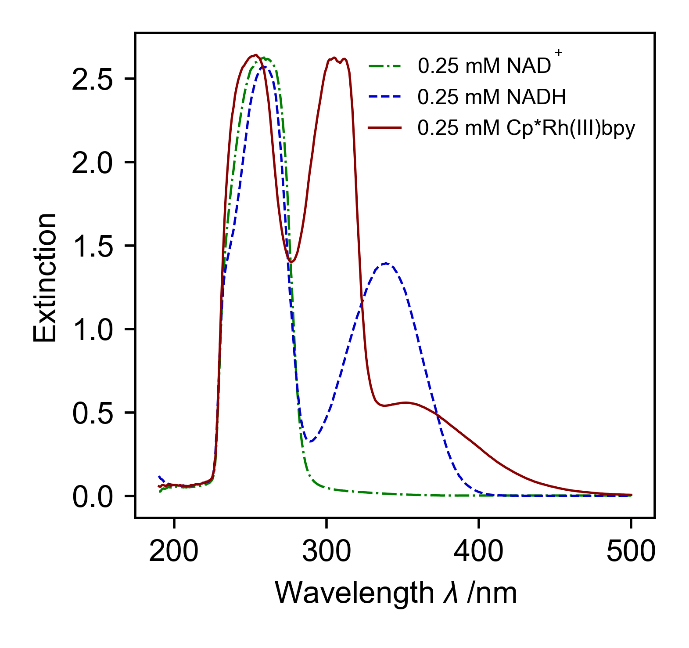


Figure 1: Extinction over the wavelength of each species in 0.1 M Hepes buffer at pH 8, without errorbars because they are to low to be shown.


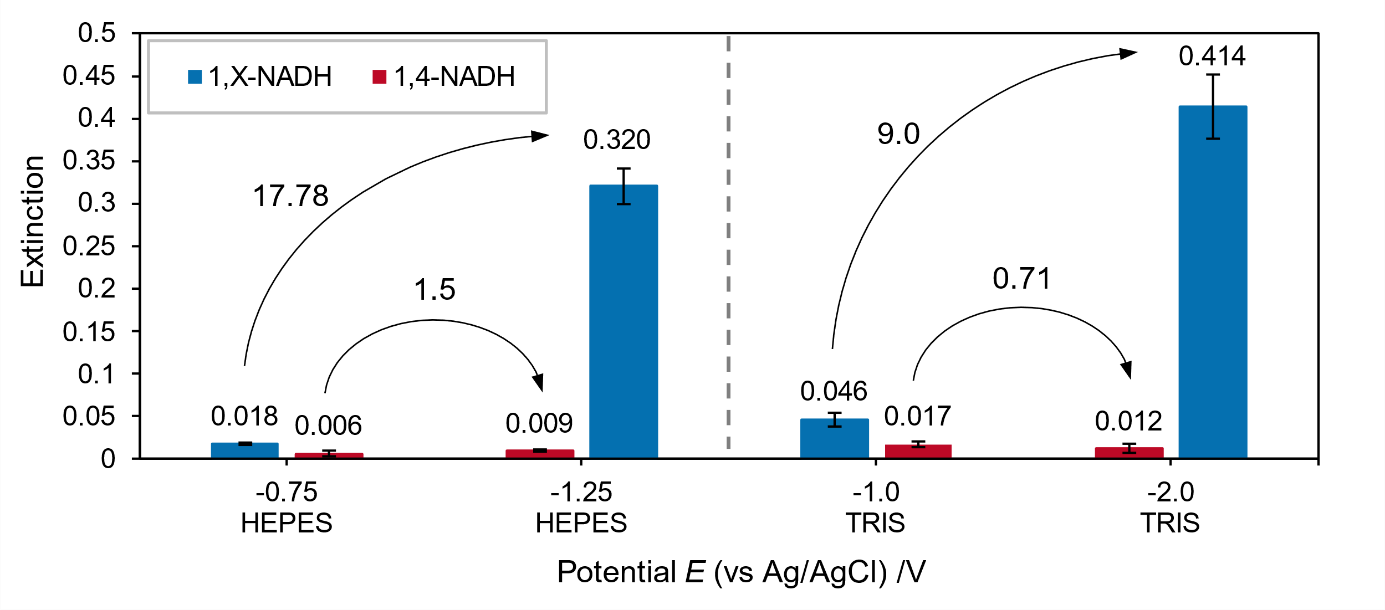


Figure 2: Direct NADH regeneration on glassy carbon in absence of rhodium based mediator in HEPES and TRIS buffer. Extinction of all NADH species and NAD_2_ declared as 1,X-NADH.
